# Ultra-low-loss on-chip zero-index materials

**DOI:** 10.1038/s41377-020-00436-y

**Published:** 2021-01-07

**Authors:** Tian Dong, Jiujiu Liang, Sarah Camayd-Muñoz, Yueyang Liu, Haoning Tang, Shota Kita, Peipei Chen, Xiaojun Wu, Weiguo Chu, Eric Mazur, Yang Li

**Affiliations:** 1 State Key Laboratory of Precision Measurement Technology and Instrument, Department of Precision Instrument, Tsinghua University, 100084 Beijing, China; 2 John A. Paulson School of Engineering and Applied Sciences, Harvard University, Cambridge, MA 02138 USA; 3 CAS Key Laboratory for Nanophotonic Materials and Devices, Nanofabrication Laboratory, CAS Center for Excellence in Nanoscience, National Center for Nanoscience and Technology, 100190 Beijing, China; 4 Department of Electronic and Information Engineering, Beihang University, 100191 Beijing, China

**Keywords:** Photonic crystals, Metamaterials, Nanophotonics and plasmonics

## Abstract

Light travels in a zero-index medium without accumulating a spatial phase, resulting in perfect spatial coherence. Such coherence brings several potential applications, including arbitrarily shaped waveguides, phase-mismatch-free nonlinear propagation, large-area single-mode lasers, and extended superradiance. A promising platform to achieve these applications is an integrated Dirac-cone material that features an impedance-matched zero index. Although an integrated Dirac-cone material eliminates ohmic losses via its purely dielectric structure, it still entails out-of-plane radiation loss, limiting its applications to a small scale. We design an ultra-low-loss integrated Dirac cone material by achieving destructive interference above and below the material. The material consists of a square array of low-aspect-ratio silicon pillars embedded in silicon dioxide, featuring easy fabrication using a standard planar process. This design paves the way for leveraging the perfect spatial coherence of large-area zero-index materials in linear, nonlinear, and quantum optics.

## Zero-index media: ultra-low loss

Calculations suggest that a new design of engineered medium can simultaneously yield a refractive index of zero and low optical loss at the telecommunications wavelength of 1550 nm. The development could lead to applications in nonlinear and quantum optics benefiting from an infinite coherence length. The approach of Tian Dong and coworkers from China and the US is to embed an array of silicon pillars (about 180 nm in radius and 1100 nm high) into a matrix of silicon dioxide to create a Dirac-cone photonic-crystal slab. Importantly, if the pillar height is chosen correctly any upwards and downwards radiation out of the slab can be made to destructively interfere thus reducing propagation loss to a level of 0.15 dB/mm. The slab should be possible to fabricate using standard processes.

## Introduction

A refractive index of zero induces a wave vector with zero amplitude and undefined direction. Therefore, light propagating inside a zero-index medium does not experience any spatial phase advance, resulting in perfect spatial coherence. This coherence can be used to demonstrate several new physics in linear, nonlinear and quantum optics, including cloaking^1^, electromagnetic energy tunnelling through a zero-index waveguide with arbitrary shape^2^, nonlinear light generation without phase mismatch^3^, lasing over a large area in single mode^4^, and superradiance of many quantum emitters over a large area^5^. A zero index can be achieved via volume plasmons provided by bulk metals around the plasma frequency^6^. However, such zero-index modes are usually associated with a large loss (short propagation length) due to the intrinsic ohmic losses of metal, especially in the optical regime.

To alleviate the ohmic losses, we can achieve a zero index based on a purely dielectric photonic crystal slab (PhC slab), supporting an accidental “Dirac-cone” degeneracy of an electric monopole mode and a magnetic dipole mode at the centre of the Brillouin zone^7^. The monopole and dipole modes correspond to zero effective permittivity and permeability, respectively, inducing an impedance-matched zero index. Dirac-cone-based zero-index PhC slabs have been implemented in both out-of-plane^8^ and in-plane^9–12^ configurations (light incident on the PhC slab perpendicular and parallel to the substrate, respectively). However, the out-of-plane configuration requires fabricating vertically stacked silicon rods, restricting the interaction region between light and the zero-index medium to a small thickness and a simple shape. In-plane Dirac-cone materials can be easily fabricated into large and arbitrarily shaped regions.

**Fig. 1:**
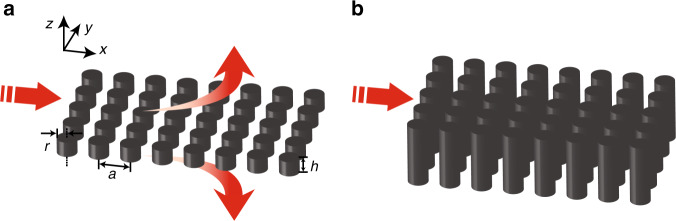
Schematic diagram of an ultra-low-loss on-chip zero-index PhC slab based on a bound state in the continuum (BIC). **a** Zero-index PhC slab without BICs whose radius, pitch and height are *r*, *a* and *h*. A photonic dipole mode forming the zero index results in out-of-plane radiation, dramatically increasing the propagation loss of the material. **b** Zero-index PhC slab with a BIC. At a particular height, all the upward/downward out-of-plane radiation destructively interferes

The in-plane configuration consists of a PhC slab, opening two radiation channels from the zero-index PhC slab upward to air and downward to the substrate. From the viewpoint of momentum conservation, these two loss channels exist because the momentum of the in-plane wave travelling within the PhC slab is less than that of the plane wave in free space, so light can couple out of the PhC slab upward and downward in the form of plane waves (Fig. 1a). From a photonic band structure viewpoint, modes forming the Dirac cone are in the region above the light line and can therefore couple to plane waves travelling perpendicular to the substrate^13^. The dipole mode, in particular, couples strongly to the out-of-plane radiation channels, giving rise to a low quality factor for this mode. The monopole and other higher-order modes, on the other hand, do not have such an out-of-plane radiation loss because of their intrinsic mode symmetry. This low out-of-plane radiation loss gives rise to a high quality factor for these symmetry-protected modes^14^.

In this paper, we present a low-loss zero-index Dirac-cone PhC slab using bound states in the continuum (BICs). In contrast to a recent demonstration^15^ of a low-loss Dirac-cone material achieved using symmetry-protected high-order modes based on boundary effective medium theory^16^, our approach involves an accidental degeneracy of a monopole mode and a dipole mode at the centre of the Brillouin zone, with the dipole mode consisting of resonance-trapped modes^17,18^. Our low-order mode-based design can be better treated as a homogeneous zero-index medium^19^ and is easier to achieve based on conventional on-chip zero-index materials^10,11^. To realize these resonance-trapped modes, we model the top and bottom interfaces of a zero-index PhC slab as two partially reflective mirrors of a Fabry-Pérot (FP) cavity (Fig. 1a) and adjust the thickness of the zero-index PhC slab to induce destructive interference in each of the radiation channels (Fig. 1b). Inside each pillar, there are axially propagating mode(s) with dipole symmetry showing a round-trip phase of an integer multiple of 2π, therefore becoming resonance-trapped modes. Because our approach involves only lower-order modes, our design results in a PhC slab that behaves as a material with *ε* = 0 and *μ* = 0 according to effective medium theory^19,20^. This approach can be applied to any array and unit-cell geometry, such as an easily fabricable square array of low-aspect-ratio silicon pillars, as long as the design supports monopole and dipole modes.

## Results

### Design and mechanism

**Fig. 2:**
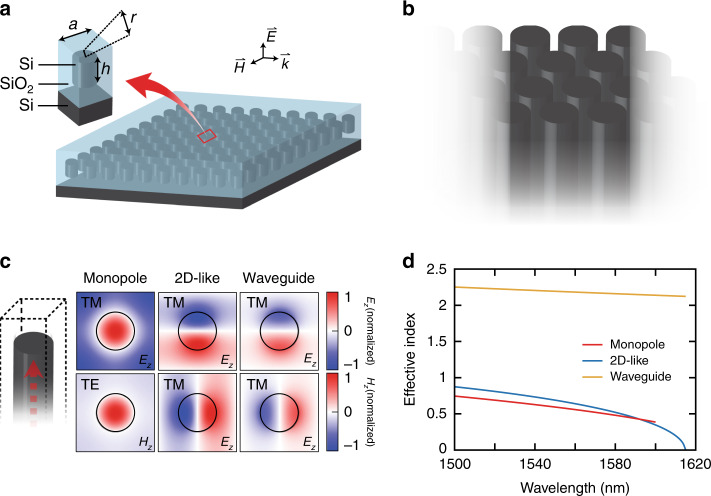
Design of a BIC zero-index PhC slab and mode analysis. **a** Three-dimensional schematic of a zero-index PhC slab and its unit cell, consisting of silicon pillars embedded in silicon dioxide with radius *r* = 180 nm, pitch *a* = 733 nm and preliminary height *h* = 1000 nm. **b** 2D array of silicon pillars. **c** This array can support six supermodes propagating along the vertical direction: TM monopole, TE monopole, and two sets of TM dipoles. Here, we plot the electric field polarized along the pillar axis for each mode, except for the TE monopole, whose axial magnetic field is shown. **d** Each mode has an effective index, derived from the propagation constant, that varies as a function of the wavelength

To design a Dirac-cone zero-index PhC slab with low out-of-plane radiation loss, we start from a conventional Dirac-cone zero-index PhC slab design^11^ consisting of a square array of silicon pillars embedded in silicon dioxide with radius *r* = 180 nm and pitch *a* = 733 nm (Fig. 2a). To analyse the modes supported by this structure, we first consider the modes supported by a square array of infinite pillars (Fig. 2b). There will be a finite set of axially propagating modes at the zero-index frequency, characterized by *k*_*z*_ ≥ 0 and *k*_*x*_ = *k*_*y*_ = 0. As shown in Fig. 2c, this structure supports six modes in the near-infrared regime that propagate along the pillar axis: one TM monopole mode, one TE monopole mode, and two sets of TM dipole modes (each set related by a 90° rotation). To evaluate the radiative coupling to free space, we only consider the dipole modes because the monopoles do not radiate in the out-of-plane direction due to their intrinsic mode symmetry. Furthermore, we can focus on a single polarization, ignoring the degenerate set with generality. This leaves two axial modes that can couple to each other and to plane waves at an interface, typically resulting in radiative loss. This situation can be regarded as a material with two distinct refractive indices. When the pillars terminate at some plane (finite pillars), these two modes will couple to each other as well as the plane wave, causing radiative loss to free space.

Figure 2d shows the effective index of each mode. The higher-index mode with dipole symmetry is labelled the “waveguide mode” due to the similarity to the fundamental dipole mode of optical fibres. The lower-index mode with dipole symmetry is called the “2D-like mode” due to its connection with the mode of the 2D zero-index material. The 2D-like mode has a cutoff wavelength of ~1615 nm, corresponding to *k*_z_ = 0, where it matches the 2D dipole mode solution. Note that this mode is not the fundamental axial mode because its cutoff frequency is greater than *f* = 0. There must be at least one other axially propagating supermode with a larger effective index—the “waveguide” mode in our case. Its refractive index can be estimated by the average index of the structure, as the mode evolves from the plane wave solution in the long-wavelength limit. The fundamental mode is guaranteed to have dipole symmetry because it couples to plane waves in the effective medium limit.

**Fig. 3:**
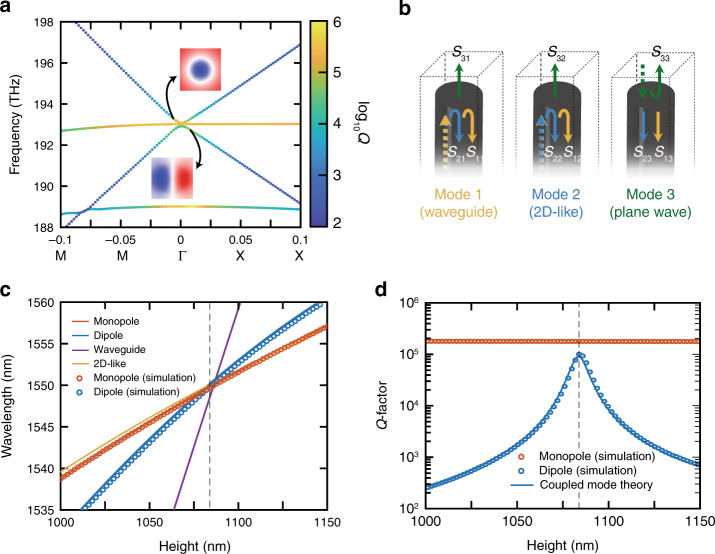
Mechanism of coupled mode theory and performance of the zero-index material calculated by both Lumerical FDTD and CMT. **a** Band structure of a zero-index PhC slab without BICs. The colormap indicates the quality factor of different modes. In the vicinity of the Dirac point, the monopole mode shows a high quality factor, while the dipole mode shows a low quality factor. **b** At the facet of the PhC slab, the waveguide and 2D-like modes can both couple to plane waves in free space, forming a three-port network. **c** Resonant wavelengths for modes of finite pillar arrays of various heights. The effective wavelength (blue) of the waveguide resonance equals the monopole resonance at pillar height *h* = 1085 nm, where *λ* = 1550 nm, forming an impedance-matched zero-index mode. **d** Radiative quality factors for modes of finite pillar arrays of various heights. In this finite pillar array structure, the Q-factor of the dipole mode shows a maximum of over 10^5^ near the BIC condition at *h* = 1085 nm, reducing the propagation loss dramatically

The low-quality-factor dipole mode shown in Fig. 3a is a hybrid mode consisting of a waveguide mode and a 2D-like mode. At the interface, any mode propagating along the axis must couple to free space or reflect back into itself or another mode of the same symmetry (Fig. 3b). Therefore, the photonic crystal slab can be treated as a three-port network formed by the waveguide mode, the 2D-like mode and the plane wave. *S*_*ij*_ (*i,j* ∈ {1,2,3}) denotes the complex coupling coefficient from mode *j* to mode *i*. There is a resonance frequency for which the waveguide mode and the 2D-like mode make a round trip with an overall phase that is a multiple of 2*π*. At this frequency, the effect of the round trip on these two modes can be described by a purely real coefficient, which accounts for the round-trip decay due to the out-coupling. For certain unit-cell dimensions, this coefficient becomes 1, and the out-coupling drops to zero, resulting in a BIC. This lossless condition can be understood as destructive interference between the waveguide mode and the 2D-like mode, forming an FP etalon.

We can define an effective Hamiltonian ***H*** for this system that accounts for the resonance-trapped mode evolution during one round trip up and down the length of the pillar “cavity”:

1 $$\begin{array}{*{20}{c}} {{\boldsymbol{H}} = \left[ {\begin{array}{*{20}{c}} {H_{11}} & {H_{12}} \\ {H_{21}} & {H_{22}} \end{array}} \right] = \left[ {\begin{array}{*{20}{l}} {S_{11}^2e^{2i\varphi _1} + S_{12}^2e^{i\left( {\varphi _1 + \varphi _2} \right)}} & {S_{11}S_{12}e^{2i\varphi _1} + S_{12}S_{22}e^{i\left( {\varphi _1 + \varphi _2} \right)}} \\ {S_{11}S_{12}e^{i\left( {\varphi _1 + \varphi _2} \right)} + S_{12}S_{22}e^{2i\varphi _2}} & {S_{12}^2e^{i\left( {\varphi _1 + \varphi _2} \right)} + S_{22}^2e^{2i\varphi _2}} \end{array}} \right]\ } \end{array}$$

where *φ*_1_ = 2*πhn*_1_/*λ* and *φ*_2_ = 2*πhn*_2_/*λ* are the phases accumulated through propagation along the pillar length *h* in each mode and *n*_1_ and *n*_2_ are the effective indices of the modes. Here, ***H*** is two-dimensional because we only consider the resonance-trapped modes for which the energy leaking to free space goes to zero. *H*_*mk*_ is the contribution coefficient of mode *k* in the previous round to mode *m* in the next round (Supplementary Section 3). Using coupled mode theory (CMT), we can directly evaluate the resonant frequencies and quality factors.

The eigenresonances of the PhC slab are given by the eigenvalues of this system,

2 $$\begin{array}{*{20}{c}} {{\boldsymbol{H}}\overrightarrow {\mathrm{v}} = \alpha \overrightarrow {\mathrm{v}} \ } \end{array}$$

where the eigenvalue *α* represents the round-trip decay. In particular, we are interested in stationary solutions where *α* is real, which only occurs at specific frequencies for a given pillar height. Figure 3c shows the resonant wavelengths (corresponding to real eigenvalues) for the eigenresonances for various pillar heights. The propagating waveguide mode and 2D-like mode couple to form the hybrid dipole mode within the pillars. Without coupling, each mode forms lossy FP resonance with different frequencies. In addition, we plot the wavelengths of the individual waveguide mode and 2D-like mode wherein we ignore the coupling between them. The hybrid dipole mode (blue) lies at a wavelength between the two uncoupled modes, which varies considerably with pillar height.

The magnitude of the eigenvalue can be translated into an effective Q-factor of the resonance-trapped mode. The Q-factor can be defined as

3 $$Q = 2\pi h\frac{{\lambda \frac{{dn}}{{d\lambda }} - n}}{{\lambda \,{\mathrm{ln}}\left( \alpha \right)}}$$

As shown in Fig. 3d, the Q-factor of the dipole mode is maximized around the same optimal height *h* = 1085 nm, at which point the radiative loss is effectively suppressed. Any residual loss is attributed to slight absorption within the silicon, as we set the imaginary part of the silicon refractive index to 2.12 × 10^−5^ to approximate experiments. Here, the eigenvalue is equal to 1 (*α* = 1) such that the eigenmode is unchanged after any number of round trips. The coupled mode predictions (solid lines) are corroborated using full-wave simulations (open circles), which confirms the accuracy of coupled mode theory.

Coupled mode theory provides necessary conditions and theoretically guarantees for the existence of bound states—first, at least two coupled modes are required in the formation of BICs. We can show that resonance-trapped BICs (lossless dipole modes) cannot exist if there is only one propagating axial mode because this precludes the existence of eigenvalues equal to 1. The round-trip phasor is equal to (1 – *r*)^2^ < 1, which means that a lossless cavity can never be formed. This also explains why we cannot obtain BICs under the cutoff frequency of the 2D-like mode. In that case, although there are two modes, the out-coupling of the 2D-like mode (*S*_32_) goes to zero.

In addition to two (or more) coupled modes, the phase condition also needs to be considered when forming BICs. This condition is always satisfied for a particular set of heights and frequencies. To see how, we can directly solve for the eigenvalues of the Hamiltonian:

4 $$\begin{array}{*{20}{c}} {\alpha = \frac{1}{2}tr\left( {\boldsymbol{H}} \right) \pm \sqrt {\frac{1}{4}tr\left( {\boldsymbol{H}} \right)^2 - det\left( {\boldsymbol{H}} \right)} \ } \end{array}$$

where *tr*(***H***) is the trace and *det*(***H***) is the determinant of the Hamiltonian. We can solve this equation directly for *α* = 1, which is the lossless case:

5 $${S_{12}^2}{e^{i({\varphi _1} + {\varphi _2})}} = \left( {1 - {S_{11}}{e^{i{\varphi _1}}}} \right)\left( {1 - {S_{22}}{e^{i{\varphi _2}}}} \right)$$

This is a condition on the wavelength-dependent scattering parameters *S*_*ij*_ (Supplementary Section 4), pillar height *h*, and propagation phase along the pillar for each mode *φ*_1_ and *φ*_2_. Note that reciprocity implies that

6 $${\left| {S_{12}} \right|^{2} = \left( {1 - \left| {S_{11}} \right|}\right)\left( {1 - \left| {S_{22}} \right|} \right)}$$

Taking the magnitude of both sides, we can see that the BIC condition is only satisfied for a particular set of phases {*φ*_1_, *φ*_2_} corresponding to a minimum for the right-hand side when both terms are real:

7 $$\left| {S_{12}^2e^{i\left( {\varphi _1 + \varphi _2} \right)}} \right| = \left| {S_{12}} \right|^2 = \left( {1 - \left| {S_{11}} \right|} \right)\left( {1 - \left| {S_{22}} \right|} \right) \le \left| {\left( {1 - S_{11}e^{i\varphi _1}} \right)\left( {1 - S_{22}e^{i\varphi _2}} \right)} \right|$$

8 $$\Rightarrow \varphi_1 = - \arg \left(S_{11} \right)$$

9 $$\begin{array}{*{20}{c}} {\varphi _2 = - {\mathrm{arg}}\left( {S_{22}} \right)\ } \end{array}$$

**Fig. 4:**
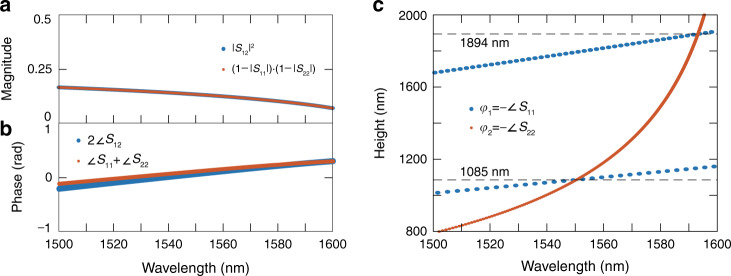
Phase conditions for BICs. **a**, **b** Magnitude and phase for the BIC condition. They are automatically satisfied for lossless/reciprocal networks containing at least two modes. **c** Conditions for half round-trip propagation phases of the two different modes. They are only satisfied at discrete points due to dispersion. BICs occur at the intersection of the two curves, which are guaranteed for discrete sets of pillar heights and effective wavelengths

In this case, the BIC condition is satisfied in both magnitude and phase for all frequencies (Fig. 4a, b, respectively). BICs occur at intersections of the resonance conditions for the two modes. There will be an infinite series of pillar heights that satisfy this condition, the shortest occurring at approximately *h* = 1085 nm (Fig. 4c).

**Fig. 5:**
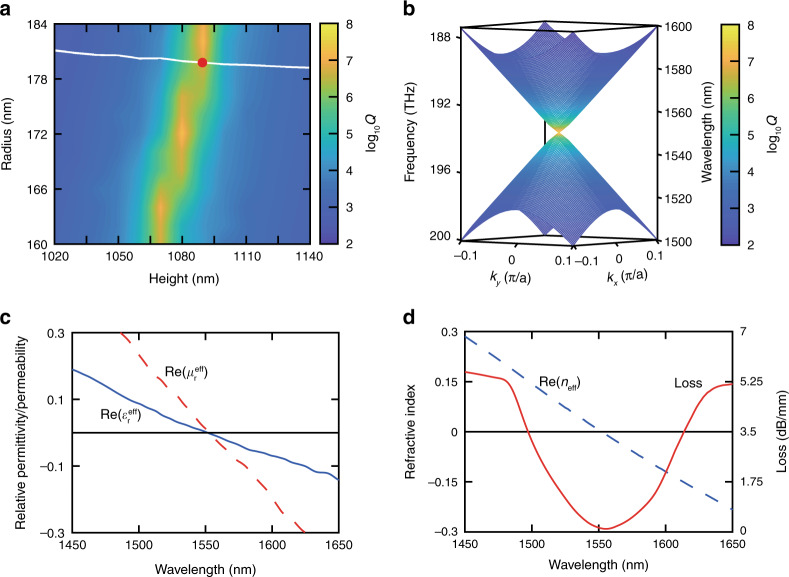
Optical properties of a BIC zero-index PhC slab. **a** Parameter sweep for design of a BIC zero-index PhC slab. Quality factor of the dipole mode (colour map) and degeneracy of monopole and dipole modes at the centre of the Brillouin zone (white line) as a function of pillar radius and height (Fig. 2a). The red dot indicates the degeneracy of a monopole mode and a high-Q dipole mode. **b** Three-dimensional dispersion surfaces showing the Dirac-cone dispersion corresponding to the optimized parameters at the red dot in (**a**). **c** Effective relative permittivity and permeability of the optimized design. Both of them linearly cross zero at 1550 nm. **d** Effective index and propagation loss of the PhC slab. When the real part of the effective index crosses zero, the loss curve reaches its valley (~0.15 dB/mm), indicating an ultra-low-loss zero index

Coupled mode theory gives us insight into the position of BICs. However, although the optimized thickness leads to a high-Q hybrid dipole mode, the accidental degeneracy of the monopole and dipole modes is broken; thus, the Dirac cone degrades to a photonic bandgap^7^. To achieve a Dirac-cone dispersion consisting of a high-Q hybrid dipole mode, we simultaneously adjust the pillar radius and the thickness of the PhC slab as two independent degrees of freedom in a narrow range (Supplementary Section 2). As shown in Fig. 5a, the quality factor of the dipole mode shows high values when the height is approximately 1080 nm and the radius ranges from 160 to 184 nm. The quality factor of the dipole is very sensitive to variations in the slab thickness, which is in good agreement with coupled mode theory. The solid white line represents the values of *r* and *h* for which the monopole mode and dipole modes are degenerate in the resulting band structure. The nearly horizontal nature of this line confirms that the accidental degeneracy is sensitive to the pillar radius but not the height^7^. The degeneracy line and the high quality-factor line are almost orthogonal to each other and cross at *r* = 180 nm and *h* = 1085 nm, as indicated by the red dot in Fig. 5a. Based on these parameters, we computed the 3D dispersion surfaces of the PhC slab shown in Fig. 5b. The result is a gapless Dirac-cone dispersion whose monopole mode and dipole mode both show high quality factors near the Dirac point.

To confirm that the Dirac-cone PhC slab can only support impedance-matched zero-index modes around the Dirac-point frequency for a wave incident normal to the interface, we compute the effective constitutive parameters in the in-plane direction. As shown in Fig. 5c, real parts of both the effective permittivity and permeability cross zero simultaneously and linearly at the design wavelength of 1550 nm, indicating an impedance-matched zero index. Furthermore, we compute the in-plane propagation loss of the material according to the retrieved effective index of refraction via

10 $$\begin{array}{*{20}{c}} {L\left[ {\frac{{{\mathrm{dB}}}}{{\mathrm{m}}}} \right] = \frac{{\omega {\mathrm{Im}}\left( {n_{{\mathrm{eff}}}} \right)}}{{4.343{\mathrm{c}}}}\ } \end{array}$$

where Im(*n*_eff_) is the imaginary part of the effective index and c is the speed of light in vacuum. As shown in Fig. 5d, the in-plane propagation loss shows a valley at the zero-index wavelength, corresponding to a BIC zero-index PhC slab with a propagation loss as low as 0.15 dB/mm. We obtain the same result when we compute the propagation loss using a cut-back method (Supplementary Section 6).

### Verification of large-area low-loss zero-index materials

**Fig. 6:**
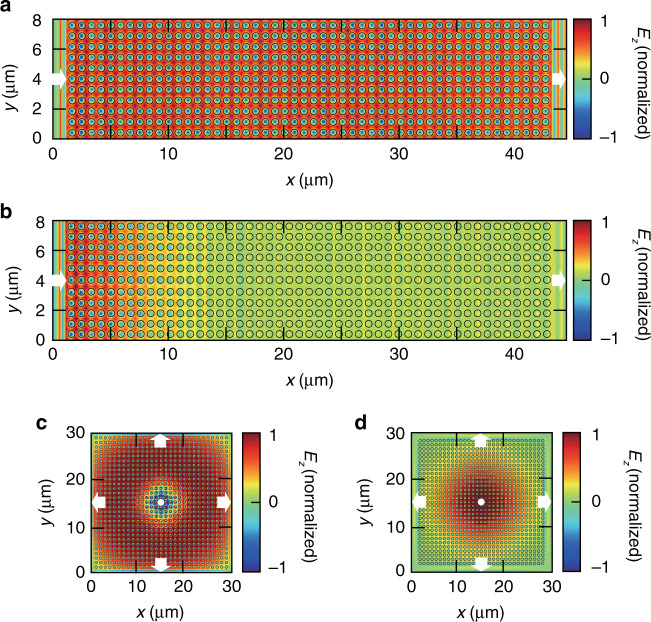
Electric field distributions over large-area zero-index PhC slabs with (a, c) and without (b, d) BICs. **a**, **b** The PhC slab is excited by a plane wave incident from the left. **c**, **d** The PhC slab is excited by an electric dipole at the centre of the material. The structural parameters of the BIC zero-index PhC slabs in (**a**, **c**) are *r* = 180 nm, *a* = 733 nm, and *h* = 1085 nm. The structural parameters of the zero-index PhC slabs without BICs in (**b**) are *r* = 180 nm, *a* = 733 nm, and *h* = 1200 nm and (**d**) are *r* = 180 nm, *a* = 733 nm, and *h* = 1247 nm

To confirm the absence of loss in a large-area BIC zero-index PhC slab, we excite zero-index PhC slabs with and without BICs using a plane wave and compare the in-plane field distributions of these two materials. As shown in Fig. 6a, the electric field distribution over the entire BIC zero-index PhC slab shows monopole-mode behaviour, corresponding to an average permittivity of zero and perfect spatial coherence. This spatial coherence enables several applications, including supercoupling^21^, zero-index phase matching^3^, and extended superradiance^22^. As shown in Fig. 6b, however, without BICs, the electric field decays sharply at the input end of the PhC slab. As a result, the BIC zero-index PhC slab is of particular significance for applications requiring a long propagation length, such as integrated optical interconnects and zero-index phase matching.

To further test the loss of a large-area BIC zero-index PhC slab excited by an interior dipole, we excite the zero-index PhC slabs with and without BICs by using a dipole source. As shown in Fig. 6c, an electric dipole at the centre of the BIC zero-index PhC slab radiates omnidirectionally over a large area. However, an electric dipole in the centre of the zero-index PhC slab without BICs can only radiate over a small area, restricting the dipole-dipole interaction distance to a small scale (Fig. 6d). In Fig. 6c, the gradual phase change from the dipole to the outside area of the material is due to the nonzero index modes at the Dirac-point wavelength. The dipole source radiates to its surrounding medium in the form of a spherical wave with an omnidirectional wave vector. This excites the off-Γ nonzero index modes near 1550 nm and therefore leads to a gradual phase change in the vicinity of the dipole source (Supplementary Section 7). For applications of zero-index PhC slabs in many-body quantum optics^5^, BIC zero-index PhC slabs ensure that all the emitters (atoms or artificial atoms) can interact with each other effectively over a large area.

## Discussion

In summary, we introduce bound states into an on-chip Dirac-cone-based zero-index material by engineering the radius and thickness of the PhC slab. Such a bound state is realized via destructive interference of the out-of-plane radiation from dipole modes forming the Dirac cone at the centre of the Brillouin zone. Our BIC zero-index PhC slab exhibits an in-plane propagation loss as low as 0.15 dB/mm at the zero-index wavelength. Furthermore, the refractive index is near zero (|*n*_eff_ | < 0.1) over a bandwidth of 4.9%. Our design methodology enables BIC zero-index PhC slabs with a Dirac-cone dispersion consisting of any modes in the multipole expansion, including monopole and dipole modes^13^.

Although this work is based on a particular pillar-array design, the same principle can be applied to a variety of photonic crystal geometries. For example, because we place no restrictions on the modes of the system that do not couple to dipole modes, we expect a similar mechanism for BICs in airhole structures that achieve zero-index propagation via dipole and quadrupole modes^10^. Furthermore, our theory could also be extended to larger systems involving more than two coupled modes. Additionally, we may be able to observe BICs in designs without mirror symmetry in the direction perpendicular to the plane of the array, such as in silicon pillar arrays placed on a silicon-on-insulator substrate. In this case, we must account for the different scattering parameters at the top and bottom interfaces.

For applications, our on-chip BIC Dirac-cone zero-index PhC slabs provide an infinite coherence length with low propagation loss. This opens the door to applications of large-area zero-index materials in linear and nonlinear optics as well as lasers, including electromagnetic energy tunnelling through a zero-index waveguide with an arbitrary shape, nonlinear light generation without phase mismatch over a long interaction length, and lasing over a large area in a single mode. This work can also serve as an on-chip lab to explore fundamental quantum optics such as efficient generation of entangled photon pairs and collective emission of many emitters. Particularly, because the spatial distribution of *E*_*z*_ in each silicon pillar oscillates between a monopole mode and a dipole mode as time elapses, all the quantum emitters within the pillars will experience the same spatial phase in the monopole half cycle. This significantly alleviates the challenge of precise positioning of quantum emitters in a photonic cavity^23^.

## Materials and methods

Our simulation is based on the frequency-domain physics of the RF module in the commercial software COMSOL Multiphysics, and the results are confirmed by Lumerical FDTD Solutions. For ease of fabrication, we design the photonic crystal slab based on a silicon-on-insulator wafer. The initial geometry parameters of the unit cell are set according to a 2D zero-index design with a radius *r* = 171.3 nm and a pitch *a* = 851 nm. The initial height of the silicon pillars is set to *h* = 1000 nm. The material parameters are based on the built-in database of COMSOL: silicon has a permittivity of 11.7 and a permeability of 1; silicon dioxide has a permittivity of 2.09 and a permeability of 1. The top and bottom boundary conditions are PMLs with a distance of half a free-space wavelength from the top and bottom of the pillars, respectively. The in-plane boundary conditions are selected as Floquet periodic boundary conditions to achieve translational symmetries in the *x* and *y* directions, corresponding to wave vectors of *k*_*Fx*_ = (*k*_*x*_·*π*)/*a*, *k*_*Fy*_ = (*k*_*y*_·*π*)/*a*, and *k*_*Fz*_ = 0. The plane wave source wavelength is selected as the optical telecom wavelength of 1550 nm. Considering the high aspect ratio of the structure, a TM-polarized source is used in the simulation.

## Supplementary information

Supplementary information

## Data Availability

The data that support the plots within this paper are available from the corresponding author upon reasonable request.

## Code availability

The code used in this paper is available from the corresponding author upon reasonable request.

## References

[1] Chu HC (2018). A hybrid invisibility cloak based on integration of transparent metasurfaces and zero-index materials. Light.: Sci. Appl..

[2] Silveirinha M, Engheta N (2006). Tunneling of electromagnetic energy through subwavelength channels and bends using ε-near-zero materials. Phys. Rev. Lett..

[3] Suchowski H (2013). Phase mismatch–free nonlinear propagation in optical zero-index materials. Science.

[4] Chua SL (2014). Larger-area single-mode photonic crystal surface-emitting lasers enabled by an accidental Dirac point. Opt. Lett..

[5] Mello, O. et al. Strongly extended superradiance in diamond metamaterials. Proceedings of CLEO: QELS_Fundamental Science 2017 (Optical Society of America, San Jose, USA, 2017).

[6] Maier, S. A. *Plasmonics: Fundamentals and Applications* (Springer, New York, 2007).

[7] Huang XQ (2011). Dirac cones induced by accidental degeneracy in photonic crystals and zero-refractive-index materials. Nat. Mater..

[8] Moitra P (2013). Realization of an all-dielectric zero-index optical metamaterial. Nat. Photonics.

[9] Li Y (2015). On-chip zero-index metamaterials. Nat. Photonics.

[10] Vulis DI (2017). Monolithic CMOS-compatible zero-index metamaterials. Opt. Express.

[11] Kita S (2017). On-chip all-dielectric fabrication-tolerant zero-index metamaterials. Opt. Express.

[12] Reshef O (2017). Direct observation of phase-free propagation in a silicon waveguide. ACS Photonics.

[13] Joannopoulos, J. D. et al. *Photonic Crystals: Molding the Flow of Light*. 2nd edn. (Princeton: Princeton University Press, 2011).

[14] Ovcharenko AI (2020). Bound states in the continuum in symmetric and asymmetric photonic crystal slabs. Phys. Rev. B.

[15] Minkov M (2018). Zero-index bound states in the continuum. Phys. Rev. Lett..

[16] Lai Y (2011). Hybrid elastic solids. Nat. Mater..

[17] Yariv, A. & Yeh, P. *Photonics: Optical Electronics in Modern Communications*, 6th edn. (New York: Oxford University Press, 2007).

[18] Saleh, B. E. A. & Teich, M. C. *Fundamentals of Photonics*, 3rd edn. (Hoboken: Wiley, 2019).

[19] Chan CT (2012). Dirac dispersion and zero-index in two dimensional and three dimensional photonic and phononic systems (Invited Paper). Prog. Electromagnetics Res. B.

[20] Wu Y (2006). Effective medium theory for magnetodielectric composites: beyond the long-wavelength limit. Phys. Rev. B.

[21] Liberal I, Engheta N (2016). Zero-index platforms: where light defies geometry. Opt. Photonics N..

[22] MacGillivray JC, Feld MS (1976). Theory of superradiance in an extended, optically thick medium. Phys. Rev. A.

[23] Jiang HT (2012). Position-independent normal-mode splitting in cavities filled with zero-index metamaterials. Opt. Express.

