# Supplementary material for: Ultra-low-loss on-chip zero-index materials
Source: Light Sci Appl. 2021 Jan 7;10:10. doi: 10.1038/s41377-020-00436-y (PMC7791033; doi:10.1038/s41377-020-00436-y)
Supplement: Supplementary file 1 — Supplementary information [file 41377_2020_436_MOESM1_ESM.docx]

Supplementary Information for

**Ultra-low-loss on-chip zero-index materials**

Tian Dong^1^, Jiujiu Liang^1^, Sarah Camayd-Muñoz^2^, Yueyang Liu^1^, Haoning Tang^2^, Shota Kita^2^，Peipei Chen^4^, Xiaojun Wu^3^, Weiguo Chu^4^,^*^ Eric Mazur^2^,^*^ and Yang Li^1^,^*^

**Affiliations:**

^1^State Key Laboratory of Precision Measurement Technology and Instrument, Department of Precision Instrument, Tsinghua University, 100084 Beijing, China.

^2^John A. Paulson School of Engineering and Applied Sciences, Harvard University, Cambridge, MA 02138, USA.

^3^Department of Electronic and Information Engineering, Beihang University, 100191 Beijing, China.

^4^Nanofabrication Laboratory, National Center for Nanoscience and Technology, 100190 Beijing, China.

**Official email addresses of all authors:**

Tian Dong: [dongt20@mails.tsinghua.edu.cn](mailto:dongt20@mails.tsinghua.edu.cn)

Jiujiu Liang: [liangjj19@mails.tsinghua.edu.cn](mailto:liangjj19@mails.tsinghua.edu.cn)

Sarah Camayd-Muñoz: camayd.munoz@gmail.com

Yueyang Liu: [liuyueyang2000@163.com](mailto:liuyueyang2000@163.com)

Haoning Tang: [hat431@g.harvard.edu](mailto:hat431@g.harvard.edu)

Shota Kita: [syouta.kita.ue@hco.ntt.co.jp](mailto:syouta.kita.ue@hco.ntt.co.jp)

Peipei Chen: [chenpp@nanoctr.cn](mailto:chenpp@nanoctr.cn)

Xiaojun Wu: [xiaojunwu@buaa.edu.cn](mailto:xiaojunwu@buaa.edu.cn)

Weiguo Chu: [wgchu@nanoctr.cn](mailto:wgchu@nanoctr.cn)

Eric Mazur: [mazur@seas.harvard.edu](mailto:mazur@seas.harvard.edu)

Yang Li: [yli9003@mail.tsinghua.edu.cn](mailto:yli9003@mail.tsinghua.edu.cn)

To whom correspondence should be addressed.

Weiguo Chu: [*wgchu@nanoctr.cn](mailto:*wgchu@nanoctr.cn), 010-82545612

Eric Mazur: [mazur@seas.harvard.edu](mailto:mazur@seas.harvard.edu), +001-(617) 495-8729

Yang Li: [yli9003@mail.tsinghua.edu.cn](mailto:yli9003@mail.tsinghua.edu.cn), +86-16601021689

**Table of Contents**

[**1.** **Relationship between monopole, dipole and the effective parameters** 3](#_Toc51253909)

[**2.** **Design procedure** 4](#_Toc51253910)

[**3.** **Deduction of the effective Hamiltonian** 5](#_Toc51253911)

[**4.** **Scattering parameters in the coupled mode theory** 6](#_Toc51253912)

[**5.** **Verification through Lumerical FDTD simulations** 7](#_Toc51253913)

[**6.** **Comparison of losses calculated via cut-back method and retrieval method** 9](#_Toc51253914)

[**7.** **Effect of non-zero index bands** 10](#_Toc51253915)

1. **Relationship between monopole, dipole and the effective parameters**

The monopole and dipole modes are two orthogonal modes in the bandstructure, corresponding to the two orthogonal components (real and imaginary parts) of the complex *E_z_*, respectively. The distribution of *E_z_* in a silicon pillar is oscillating between a monopole mode and a dipole mode as time increases. In the monopole case, as shown in Figure S1a, by carefully engineering the radius and pitch of the photonic crystal slab, the polarization $\vec{P}$ will finally cancel the applied *E_z_* field, resulting in a zero electric displacement according to $\vec{D}=\varepsilon_{0}\vec{E}+\vec{P}=\varepsilon_{\mathrm{eff}}\vec{E}=0$. This gives rise to a zero effective permittivity $\varepsilon_{\mathrm{eff}}$. Similarly, in the dipole case, there will be two polarizations, one pointing upward while the other pointing downward as shown in Figure S1b. These two polarizations form a loop, and therefore induce a magnetization $\vec{M}$ with an opposite direction against the applied magnetic field according to Faraday's law. After carefully tuning the pitch and radius, the magnetization $\vec{M}$ will cancel out the applied magnetic field according to $\vec{B}=\mu_{0}(\vec{H}+\vec{M})=\mu_{\mathrm{eff}}\vec{H}=0$, which results in a zero effective permeability $\mu_{\mathrm{eff}}$.


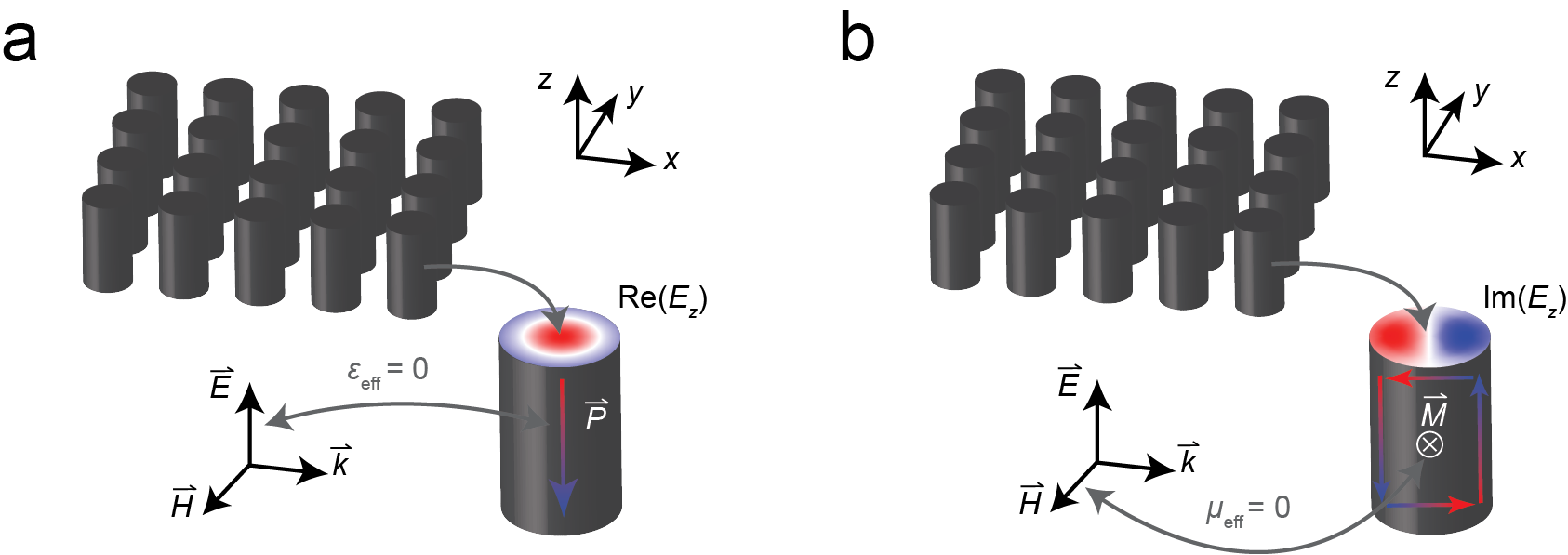


**Figure S1|** Relationship between the electric monopole, the transverse magnetic dipole and the effective parameters. **a** At the centre of the monopole mode, the polarization $\vec{P}$ always has the opposite phase against the applied *E_z_* field. The cancellation between $\vec{P}$ and $\vec{E}$ results in a zero electric displacement and thus a zero effective permittivity $\varepsilon_{\mathrm{eff}}$. **b** The dipole induces two polarizations with different directions inside the dielectric pillar, forming a loop of electric field. A magnetization $\vec{M}$ with an opposite phase against the applied magnetic field will appear according to Faraday’s law. The magnetization cancels out the incident magnetic field for certain unit-cell dimensions, which in turn causes a zero effective permeability $\mu_{\mathrm{eff}}$.

1. **Design procedure**

To design a low-loss zero-index material, we start from a zero-index photonic crystal slab (PhC slab) consisting of a square array of high aspect-ratio silicon pillars embedded in silicon dioxide. The height of the pillars determines the round-trip phases of different modes, therefore becoming the most important parameter in the design of BIC. Different pillar heights correspond to different eigenmode solutions of the Maxwell equations. These modes feature different Q-factors, enabling us to optimize the height by selecting the modes with the highest Q-factors. As for the design of zero index, the radius and pitch of the photonic crystal slab are the two main parameters determining the existence of a Dirac cone. As a result, we obtain a zero-index PhC slab with an initial thickness by engineering the radius and pitch. We then optimize the height of the silicon pillars to search for BIC modes near the Dirac-point frequency. Finally, we fine tune the radius and height to achieve a Dirac cone consisting of BIC modes.


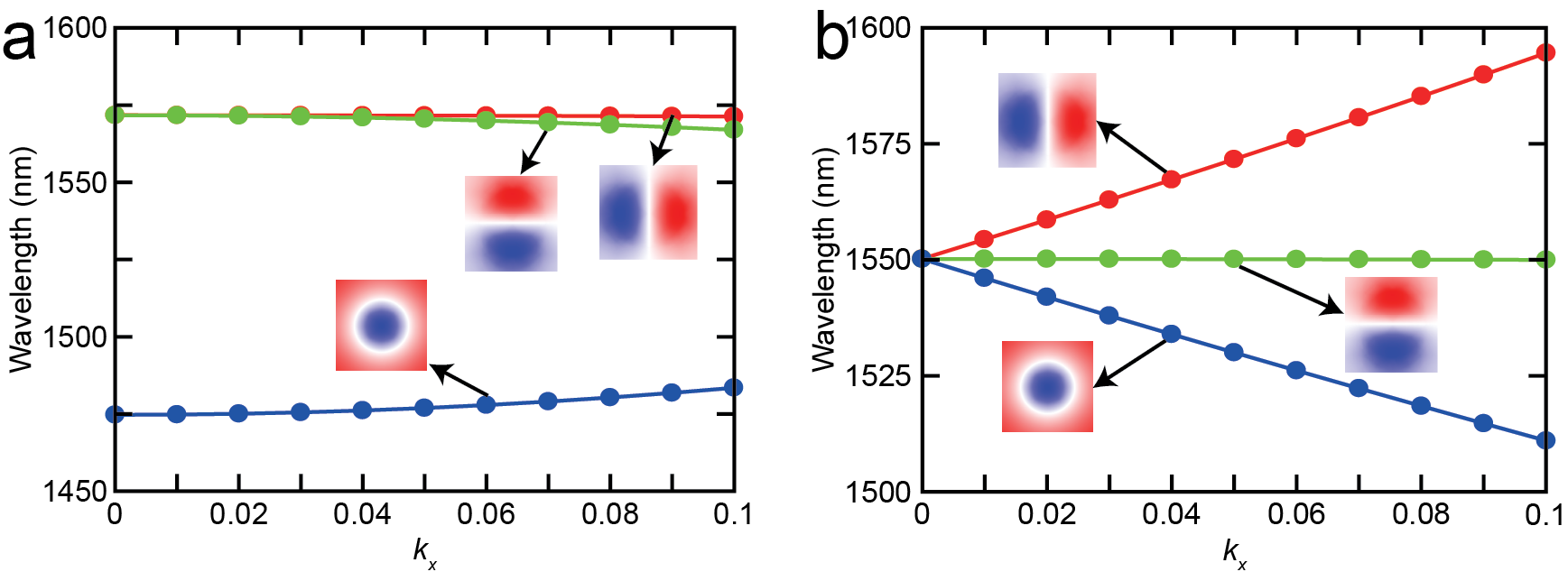


**Figure S2|** Bandstructure of the photonic crystal slab before and after engineering the radius and pitch of the pillars. **a** There is a band gap between the electric monopole and the magnetic dipoles with the initial parameters: *r* = 171 nm, *a* = 851 nm. **b** A Dirac cone is formed in the centre of the Brillouin zone. The electric monopole and the magnetic dipoles are degenerated at the operating wavelength of 1550 nm.

With the initial geometry parameters of 171-nm radius and 851-nm pitch, there is a ~97-nm photonic bandgap between the electric monopole and the magnetic dipoles at the Γ point as shown in Figure S2a. By changing the radius and pitch of the photonic crystal slab, we obtain the following variation rule of the band gap and degenerate frequency.

(1) When the radius of the pillars is fixed, the eigenwavelengths of the three modes increase as the pitch increases. Moreover, the eigenwavelength of monopole increases faster than that of dipoles. Because the initial eigenwavelength of the monopole is shorter than that of the dipoles (Figure S2a), these three modes would degenerate as we increase the pitch.

(2) When the pitch of the photonic crystal slab is fixed, the eigenwavelengths of three modes increase as the radius increases. Furthermore, the eigenwavelength of the dipoles increases faster than that of the monopole.

According to the above design rules, we begin with a structure showing the bandstructure in Figure S2a. We first fix the pitch and decrease radius to achieve a degeneracy of monopole and dipole modes at a wavelength shorter than 1550 nm. Then, we adjust the pitch and radius simultaneously to move the triply degenerated Dirac cone to 1550 nm. This preliminary design has a radius of 180 nm and a pitch of 733 nm (Figure S2b).

To see the effect of height on the Q-factor of the transverse magnetic dipole mode of the preliminary design (*r* =180 nm, *a* =733 nm), we scan the height from 10 to 1200 nm at a step of 10 nm. In each scan step, we compute all the modes supported by the photonic crystal slab from 1450 to 1650 nm at the centre of the Brillouin zone. To obtain a Dirac-cone dispersion consisting of TM-polarized modes, we select modes with the ratio between *z* component of electric field and the electric field intensity (TM fraction) over 50%. From those modes, we get a monopole mode which always shows a high Q-factor and a magnetic dipole mode showing a high Q-factor when the height is ~1085 nm. Based on the preliminarily optimized design, we choose the radius and height as two degrees of freedom, with which we perform a 2D parameter sweep to achieve a Dirac cone consisting of BIC modes. This result can be found in Fig. 5a.

1. **Deduction of the effective Hamiltonian**

In the case of resonance-trapped modes, we can directly evaluate the resonant frequencies and quality factors using coupled mode theory: first, represent the hybrid dipole mode by the vector [*v*_1_, *v*_2_]^T^, whose elements correspond to the relative amplitude circulating in each of the two axially propagating modes within the pillar. Essentially, the photons in each mode can take one of two paths in any round trip (Figure S3). This provides four ways to form the new mode 1—ICBI, IDBI, IICBI, IIDBI. Similarly, the new mode 2 can be attributed to ICAII, IDAII, IICAII, IIDAII. Assume that Point I and II are the start points of the two propagating modes in a round trip. At the interface, the photons in each mode can either transfer to another mode or stay in the same mode. Therefore, the new hybrid dipole mode after a round trip can be written as

$$\begin{aligned} \left[ \begin{aligned} V_{1}^{'} \\ V_{2}^{'} \end{aligned} \right]=\left[ \begin{matrix} S_{11}e^{i\varphi_{1}}S_{11}e^{i\varphi_{1}}+S_{21}e^{i\varphi_{2}}S_{12}e^{i\varphi_{1}} & S_{12}e^{i\varphi_{1}}S_{11}e^{i\varphi_{1}}+S_{22}e^{i\varphi_{2}}S_{12}e^{i\varphi_{1}} \\ S_{11}e^{i\varphi_{1}}S_{21}e^{i\varphi_{2}}+S_{21}e^{i\varphi_{2}}S_{22}e^{i\varphi_{2}} & S_{12}e^{i\varphi_{1}}S_{21}e^{i\varphi_{2}}+S_{22}e^{i\varphi_{2}}S_{22}e^{i\varphi_{2}} \end{matrix} \right]\cdot\left[ \begin{aligned} V_{1} \\ V_{2} \end{aligned} \right]=H\cdot\left[ \begin{aligned} V_{1} \\ V_{2} \end{aligned} \right]\#\left( AUTONUM \backslash* Arabic \right) \end{aligned}$$

where ***H*** is the effective Hamiltonian for this system that accounts for the resonance-trapped modes evolution during one round trip up and down the length of the pillar “cavity”, and it can be simplified to

$$\begin{aligned} \boldsymbol{H}=\left[ \begin{matrix} {S_{11}}^{2}e^{2i\varphi_{1}}+{S_{12}}^{2}e^{i\left( \varphi_{1}+\varphi_{2} \right)} & S_{11}S_{12}e^{2i\varphi_{1}}+S_{12}S_{22}e^{i\left( \varphi_{1}+\varphi_{2} \right)} \\ S_{11}S_{12}e^{i\left( \varphi_{1}+\varphi_{2} \right)}+S_{12}S_{22}e^{2i\varphi_{2}} & {S_{12}}^{2}e^{i\left( \varphi_{1}+\varphi_{2} \right)}+{S_{22}}^{2}e^{2i\varphi_{2}} \end{matrix} \right] \boldsymbol{\#}\left( AUTONUM \backslash* Arabic \right) \end{aligned}$$

due to reciprocity. Here $\varphi_{1}=2\pi hn_{1}/\lambda$ and $\varphi_{2}=2\pi hn_{2}/\lambda$ are the phases accumulated through propagation along the pillar length *h* in each mode, *n*_1_ and *n*_2_ are the effective indices of the modes, and *λ* is the operating wavelength.


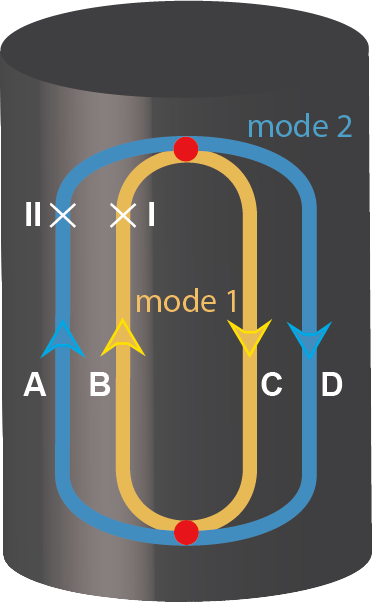


**Figure S3|** Round trip model for mode propagation inside the pillar. At the interface (denoted by the red dots), the photons in one mode can stay in the same mode or transfer to another one. This provides four possibilities in the formation of the new waveguide/2D-like mode.

1. **Scattering parameters in the coupled mode theory**

We can obtain the complex scattering parameters of this three-port network using COMSOL numerical simulations. Along with the propagation constants *k_i_* of the modes, the coupling coefficients define a set of FP resonances in a finite waveguide array. As shown in Figure S4a and b, the scattering parameters vary continuously as a function of operating wavelength. Considering the network is reciprocal and lossless, the scattering parameters are constrained by Stokes relations and energy conservation. We then use two metrics—reciprocity and passivity—to testify the validity of the retrieved *S*-parameters (Figure S4c and d). The parameters in *S* matrix which are located at the symmetrical positions with respect to the diagonal (*S_ij_* and *S_ji_*) have exactly the same value. This is consistent with the reciprocity metric, thus confirming the correctness of the *S*-parameters. For the passivity condition, we should have

|*S*_11_|^2^ + |*S*_12_|^2^ + |*S*_13_|^2^ = 1

|*S*_21_|^2^ + |*S*_22_|^2^ + |*S*_23_|^2^ = 1

|*S*_31_|^2^ + |*S*_32_|^2^ + |*S*_33_|^2^ = 1

*S*_11_*S*_21_^*^ + *S*_12_*S*_22_^*^ + *S*_13_*S*_23_^*^ = 0

*S*_11_*S*_31_^*^ + *S*_12_*S*_32_^*^ + *S*_13_*S*_33_^*^ = 0

*S*_21_*S*_31_^*^ + *S*_22_*S*_32_^*^ + *S*_23_*S*_33_^*^ = 0

This is validated in Figure S4d, making the calculated *S* parameters authentic.


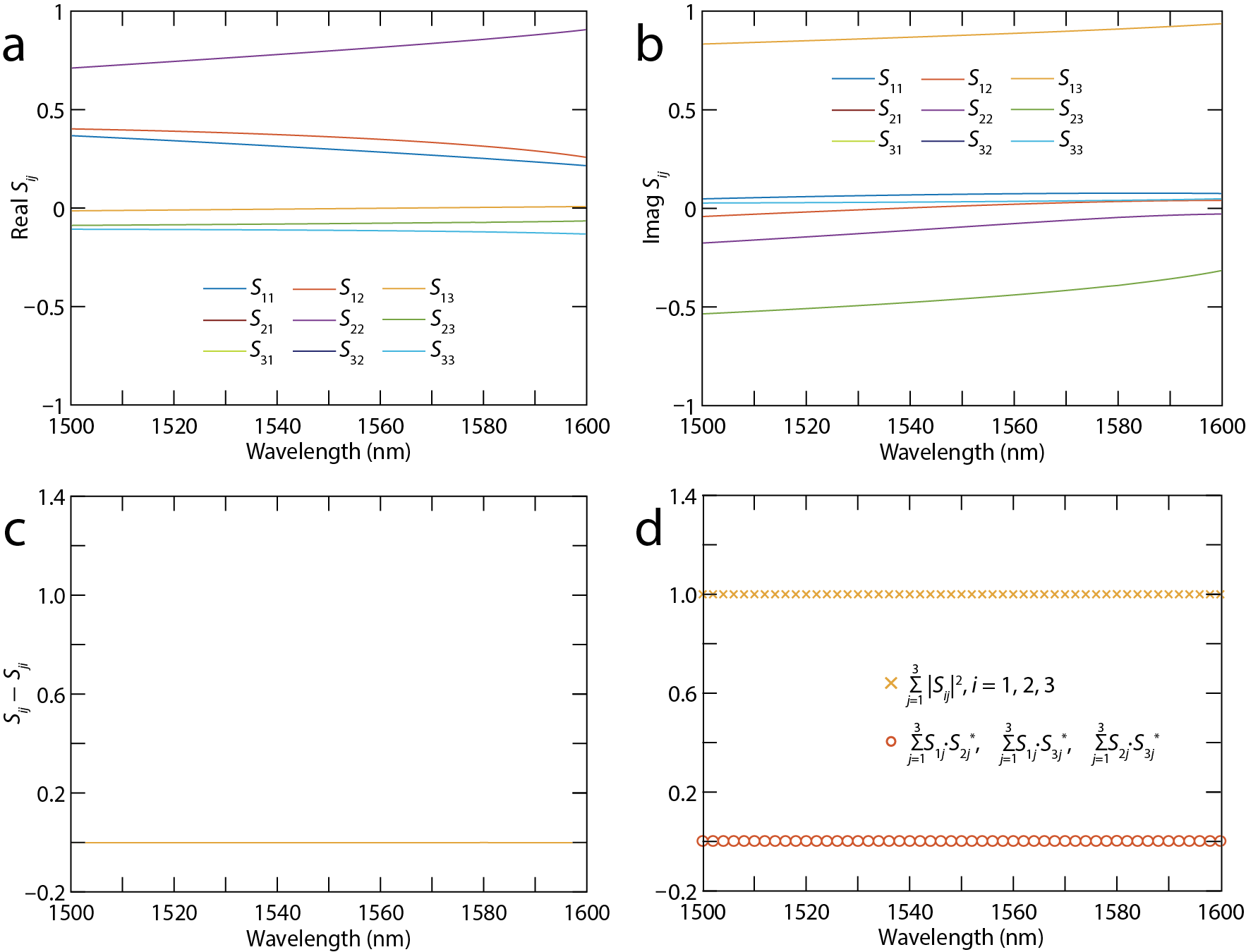


**Figure S4|** Scattering parameters of the three-port network formed by the waveguide mode, the 2D-like mode and the plane wave (Fig. 2c). **a-b** The real and imaginary part of the 9 scattering parameters. *S*_12_ and *S*_21_, *S*_13_ and *S*_31_, *S*_23_ and *S*_32_ overlap with each other. **c-d** Validation of the retrieved scattering parameters using reciprocity and passivity conditions.

1. **Verification through Lumerical FDTD simulations**

To confirm the reliability and stability of our design, we further use Lumerial FDTD to prove the effectiveness of the model. As shown in Figure S5, we first build the model of photonic crystal slab using the optimal parameters obtained in COMSOL, and add a silicon dioxide waveguide in *x*-direction. The boundary condition in *y*-direction is set to be periodic Bloch condition, and boundary conditions in *x*- and *z*-directions are PML. The system is excited by a TM mode source on the left side.


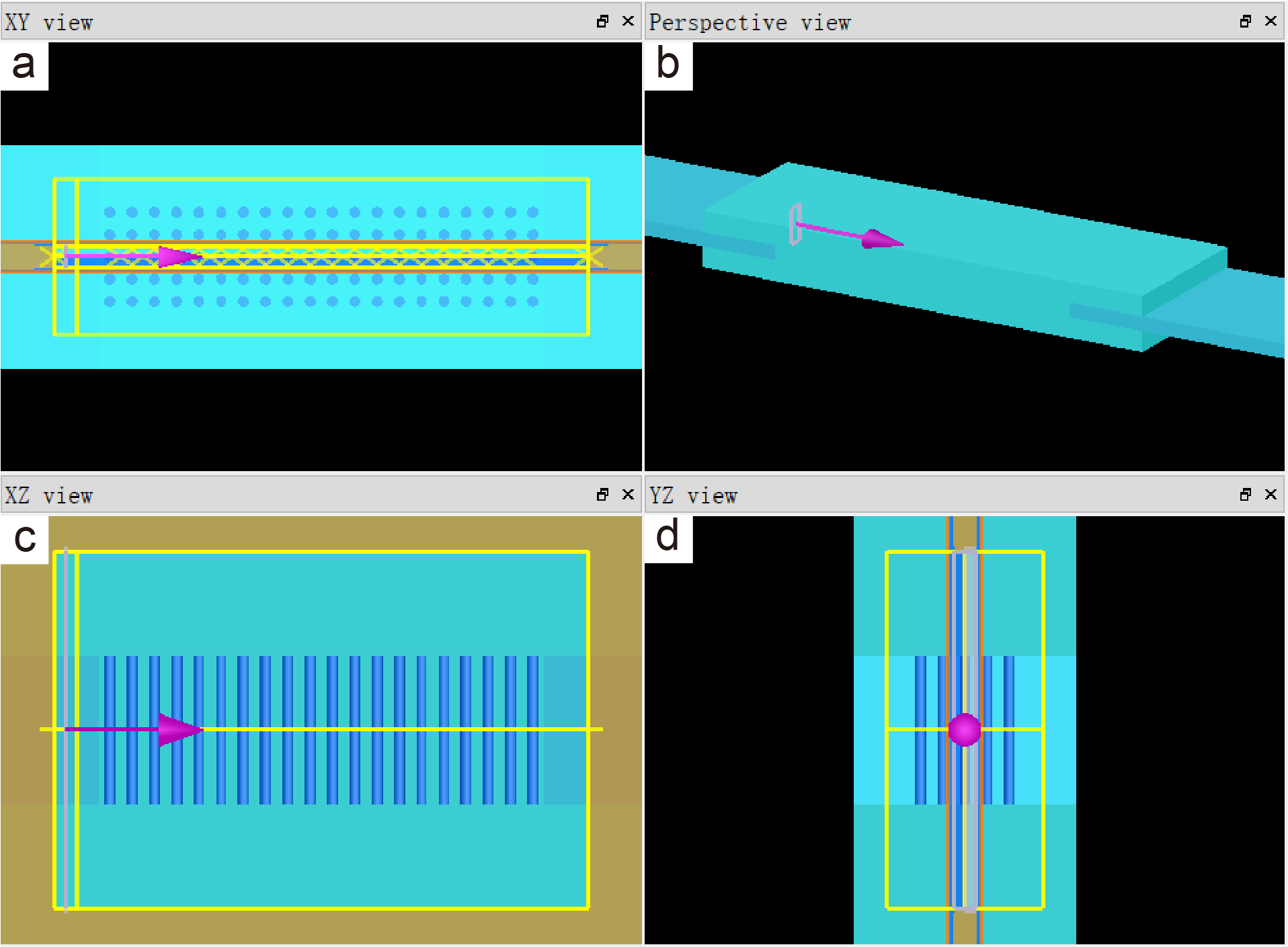


**Figure S5|** FDTD model of zero-index photonic crystal slab. **a-d** are XY-view, Perspective view, XZ-view and YZ-view, respectively.

We then calculate the in-plane transmission of the photonic crystal slab. As shown in Figure S6, the transmission along *x*-direction shows a maximum of 90% at the design wavelength 1550 nm, validating the effectiveness of low-loss zero-index PhC slab.


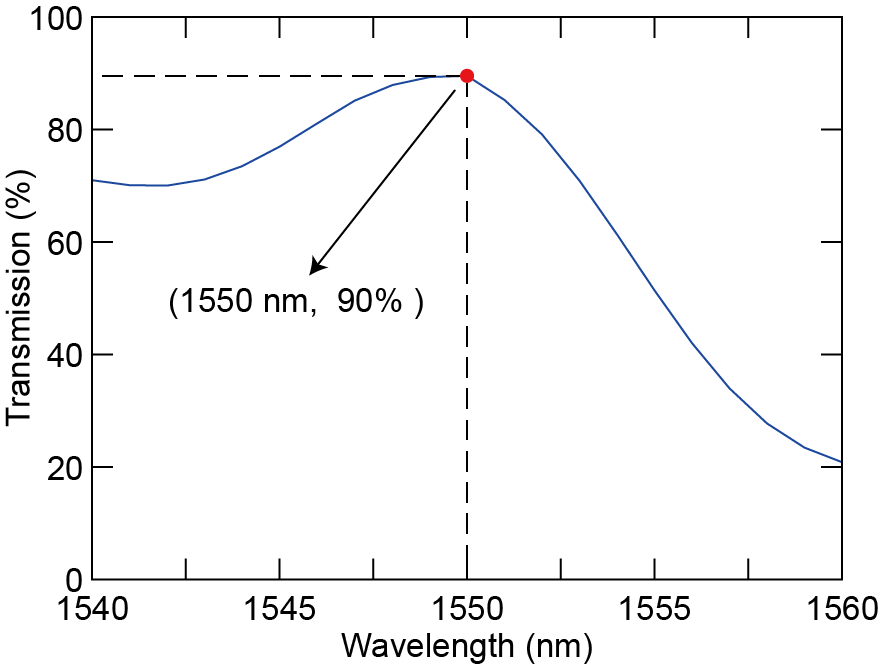


**Figure S6|** Transmission through the YZ-plane versus wavelength. The transmission curve shows a peak value of 90% at operating wavelength, 1550 nm, as denoted by the red dot.

1. **Comparison of losses calculated via cut-back method and retrieval method**

To validate the propagation loss calculated by retrieval method in the main text, we use cut-back method[^1^](#_ENREF_1) to compute the propagation loss. As shown in Figure S7a, by monitoring the transmission at the output port of the zero-index PhC slabs with different lengths, we obtain the propagation loss by calculating the slope of the transmitted power versus waveguide length line. Results show that these two methods agree with each other well, especially near the zero-index wavelength, 1550 nm (Figure S7b). Both retrieval and cut-back methods give lower loss per unit length for longer zero-index PhC slabs. When the number of unit cells along the propagation direction is less than ~200, the cut-back method gives lower loss per unit length than that of the retrieval method. When the material is longer than ~200 unit cells, cut-back and retrieval methods give similar results, especially around the zero-index wavelength.


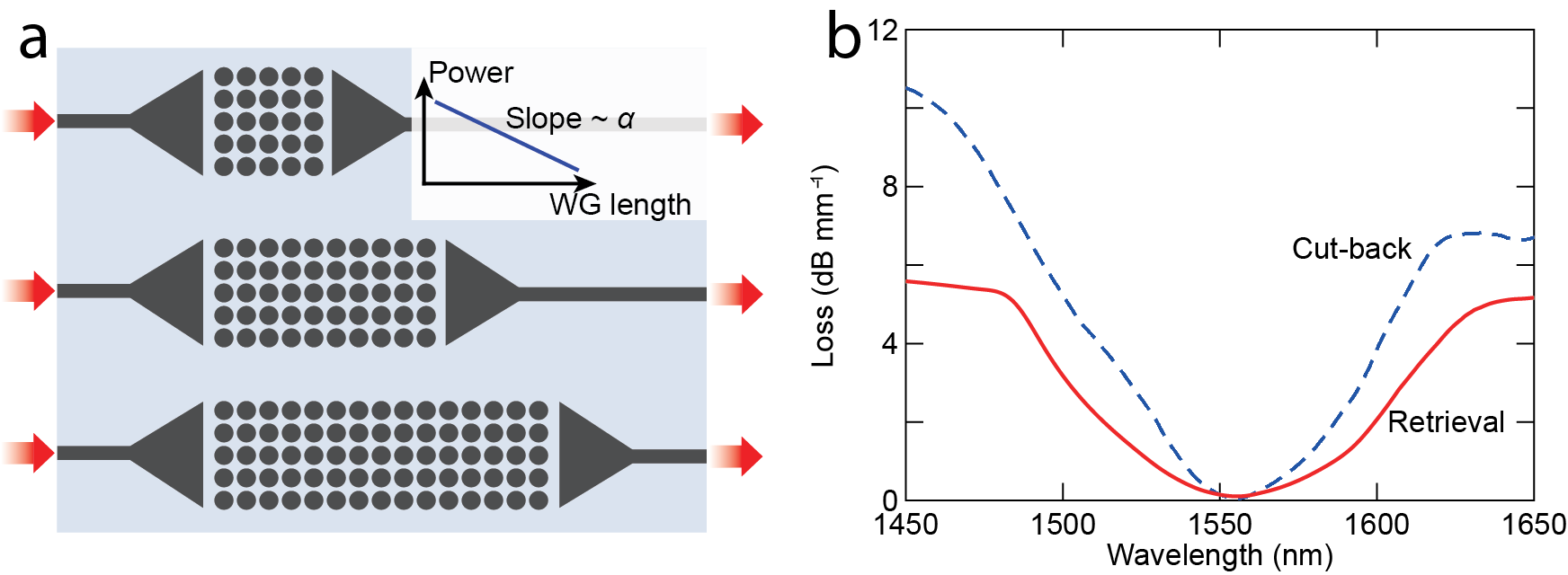


**Figure S7|** Computing propagation loss of the zero-index PhC slab by using cut-back method. **a** Schematic diagram for computing propagation loss of the zero-index PhC slabs using cut-back method. At least five pillars in the transverse direction are required to meet the homogenization criteria. **b** Comparison of the losses computed by cut-back method and that computed by retrieval method. The raw data of the loss spectra show ripples near the zero-index wavelength because the interference of multiple reflections in the Fabry-Pérot cavity formed by the input and output interfaces of the zero-index PhC slab. To reduce these ripples, we postprocess the loss spectra using the ‘smooth’ function in MATLAB.

1. **Effect of non-zero index bands**

In Fig. 5c, the phase gradually changes from the dipole source to the outside area of the material along the radial direction. Such a phenomenon is caused by the non-zero index modes near the Dirac-point wavelength (1550 nm). As shown in Figure S8, two bands degenerate with each other at the Γ point near 1550 nm, leading to an impedance matched zero-index. However, away from the Γ point, there exist some non-zero index bands near 1550 nm. When those non-zero index modes are excited, the field within the photonic crystal slab does not show the typical zero-index behavior—oscillating between a monopole mode and a dipole mode. In Fig. 5c, the system is excited by a vertical electrical dipole which radiates spherical wave in the near-field region. Such a radiation’s wave vector is aligned in all directions in the plane of the photonic crystal slab, corresponding to the large wave vector of the bandstructure, in turn excite the off-Γ non-zero index modes near 1550 nm. Those non-zero index modes lead to the gradual phase change of *E_z_* from the dipole position toward the outside area of the material (Fig. 5c).

When the distance from the dipole increases, the spherical wave gradually becomes a plane wave according to the far-field region criterion $R>2D^{2}/\lambda$, where *R* is the distance from the dipole source, *D* is the largest dimension of the dipole source and $\lambda$ is the operating wavelength[^2^](#_ENREF_2). Because a plane wave only excites the modes corresponding to the zero wave vector at Γ point, *E_z_* in the region further away from the dipole source shows a typical zero-index behavior at 1550 nm (Fig. 5c). In Fig. 5d, the gradual phase change behavior is much less apparent than that in Fig. 5c due to the large propagation loss of the zero-index modes and non-zero index modes. The propagation loss of non-zero index modes (with a Q-factor~10^2^) is larger than that of zero-index modes (with a Q-factor~10^3^), leading to a fast decaying zero-index field distribution without apparent gradual phase change.


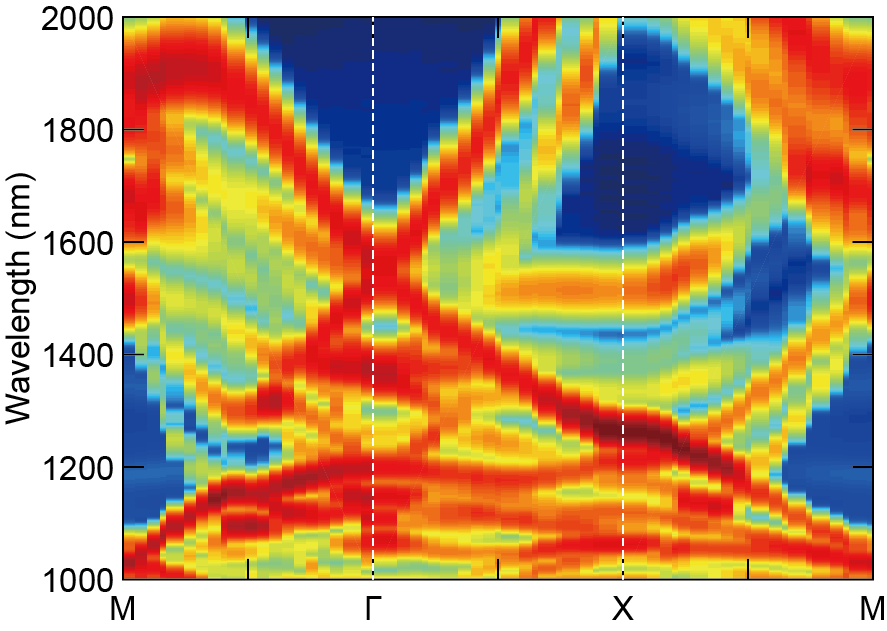


**Figure S8|** Bandstructure of the three-dimensional zero-index photonic crystal slab computed by Lumerical FDTD Solutions. There is a Dirac cone consisting of two linear bands at the Γ point, giving arise to effective zero permittivity and permeability. Away from the Γ point, there appear some non-zero index bands, inducing the gradual phase change phenomenon near the dipole source (Fig. 5c).

**References**

[1] Qi, Y. & Li, Y. Integrated lithium niobate photonics. *Nanophotonics* (2020).

[2] Balanis, C. A. Antenna theory: analysis and design (John Wiley & Sons, 2016).
